# Preferences and end of life care for residents of aged care facilities: a mixed methods study

**DOI:** 10.1186/s12904-023-01239-9

**Published:** 2023-09-01

**Authors:** Moberley Sarah, Hewitt Jacqui, Attia John, Cole Janean, Bevington Joelle, Oldmeadow Christopher, Howard Zach, Hughes Rachel

**Affiliations:** 1 Department of Palliative Care, Calvary Mater Newcastle, Newcastle, NSW Australia; 2 Faculty of Health and Medicine, University of Newcastle, Newcastle, NSW Australia; 3 Hunter Medical Research Institute, Newcastle, NSW Australia; 4 Anglican Care, Booragul, NSW Australia

**Keywords:** Nursing Home Care, Terminal Care, Symptoms and Symptom Management

## Abstract

**Background:**

Residential aged care facilities is one of the most common places to deliver of end of life care. A lack of evidence regarding preferred place for end of life care for residents of aged care facilities impacts on delivery of care and prevents assessment of quality of care. This paper reports the preferences, current status of end of life care and enablers and barriers of care being delivered in line with the wishes of residents of participating aged care facilities.

**Methods:**

We collaborated with six equally sized aged care facilities from the Greater Newcastle area, New South Wales, Australia. An audit of the quality of end of life care for residents was conducted by retrospective medical record review (n = 234 deceased patients). A retrospective review of emergency department transfers was conducted to determine the rate of transfer and assign avoidable or not. Qualitative focus group and individual interviews were conducted and analysed for barriers and enablers to end of life care being delivered in accordance with residents’ wishes.

**Results:**

Most residents (96.7%) wished to remain in their residential aged care facility if their health deteriorated in an expected way. Residents of facilities whose model of care integrated nurse practitioners had the lowest rates of emergency department transfers and timelier symptom management at end of life. Family decision making influenced location of death (either supporting or preventing care in place of patient preference).

**Conclusion(s):**

To better provide care in accordance with a person’s wishes, aged care facilities need to be supported to enable end of life care insitu through integrated care with relevant palliative care providers, education and communication strategies. Family and community health and death literacy interventions should accompany clinical innovation to ensure delivery of care in accordance with residents’ preferences.

**Supplementary Information:**

The online version contains supplementary material available at 10.1186/s12904-023-01239-9.

## Background

Managing end of life is core to the care provided in residential aged care facilities, even though this location is the least preferred by Australians surveyed for their end of life [1]. Having end of life care delivered in a preferred place is one component of ‘a good death’ [2]. There is however no available evidence for the preferences for end of life care of Australians currently residing in such facilities and little known about their end of life experience.

In Australia, approximately 242,774 people reside in permanent residential aged care [3]. A significant proportion do not receive end of life care in place, with 28.5% of people on hospital leave within 30 days of death and 14.1% dying whilst on hospital leave [4]. Such hospital transfers may not be aligned with preference and may be associated with discomfort and unnecessary medical intervention [5].

The feasibility of being cared for in the aged care facility in alignment with preference is limited by the resources available. Common reasons for avoidable transfers to emergency department include staff members of the Residential Aged Care Facility (RACF) electing for hospital transfer, lack of availability of a registered nurse overnight, resulting in limitations for treatments, and decisions around transfer being deferred to less qualified staff, influenced by family preference [6].

With increasing life expectancy and improved management of chronic illness, many Australians have limited exposure to death. A recent death literacy survey in Australia showed personal experience was the highest-ranking factor contributing to knowledge about death and dying [7]. As such, limited experience and uncertainty with terminal illness may hamper substitute decision makers from supporting end of life care consistent with residents’ preferences.

For those residents that remain in place for end of life, care may be less than optimal. The quality of this care is of critical concern for the community [8]. The interim findings of an Australian Royal Commission have reported “patchy and fragmented palliative care for residents who are dying, creating unnecessary distress for both the dying person and their family.”[9].

Decision making for aged care facilities around hospital transfers at the end of life is dependent upon various factors including medical practitioner availability, nursing staff levels, and advance care planning processes [6].

There is evidence that structural characteristics of RACFs, including ownership, funding, size, and affiliations also determine the quality of end of life care [10]. There is minimal comparative research assessing quality outcomes for the aged care resident in order to identify the optimal model to support end of life care in place and how specialised palliative resources can be best utilised by facilities [11]. Available evidence describes effective components of models, including integrated palliative care leadership, symptom control strategies, and communication strategies [12].

Novel approaches to enabling end of life care in RACFs are emerging, with evidence to support the cost effectiveness of proactive specialist palliative clinical and enablement input into RACFs, reducing length of stay for those RACF residents who are transferred to acute hospital [13].

To better understand optimal, patient-centred delivery of end of life care in RACFs, a project was undertaken to address the following research questions:

- What are the documented preferences and experiences of end of life care for residents of aged care facilities using 3 different models of palliative care provision,
- Describe the rate and avoidability of ED presentations according to facility type,
- Determine whether the different models of care within the RACF affect residents’ experiences.

## Methods

The study took place in Newcastle, New South Wales, Australia, a large regional city with a comprehensive palliative care system including a public hospice, private palliative care wards, publically-funded palliative care outreach teams, and a large number of aged care facilities run by a variety of not-for-profit and private organisations. Purposive sampling of six facilities with 50 to 100 residents were recruited based on their level of engagement with Specialist Palliative Care:

A. Two RACFs with little or no Specialist Palliative Care engagement (care led by General Practitioners).
B. Two RACF’s with regular engagement with Specialist Palliative Care outreach service.
C. Two RACFs who employ a nurse practitioner as part of their routine of care. A nurse practitioner in Australia has an autonomous role within a nursing speciality, in this instance, palliative care. Importantly, nurse practitioners are authorised to prescribe medications.

We conducted an assessment of factors pertaining to end of life care utilising mixed methods to capture the preferences, end of life experiences and the underlying reasons for care being delivered in alignment with preferences:

### Preferences and care at end of life for aged care residents

A list of deceased residents from each facility for 2017 and 2018 was obtained. The medical records of all deaths were reviewed if less than 50 had occurred within that period, or a random selection of up to 50 were reviewed if over 50 deaths had occurred. We utilised the End of Life Directions for Aged Care After Death Audit Tool [14] to assess: the preferred location residents wished to be cared for at end of life; goals of care; and the presence and management of symptoms at end of life. In particular, the preferred place of end of life care was determined from patient notes or based on the Medical Order for Life Sustaining Treatment (documentation of no attempt for cardiopulmonary resuscitation – accept natural dying, palliative care during natural dying and avoid hospital transfer). Some components of the audit tool were not collected as they were out of scope.

### Review of rate and avoidable classification for ED presentations

Data on public hospital ED presentations for residents were collected during the same two-year period. Where a patient was transferred from one public hospital to another public hospital, the initial presentation was excluded. Presentations were categorised as avoidable or non-avoidable by a Specialist Palliative Care physician who was blinded to the facility. Avoidable presentations were defined as those with clear symptomatology which could be reasonably diagnosed and managed within a non-hospital facility staffed at the level of a registered nurse with General Practitioner / nurse practitioner availability for direct or indirect clinical assessment and prescribing. Non avoidable transfers included unexpected deteriorations requiring hospital treatment including fall and fracture and acute onset, unexpected clinical deteriorations such as chest pain or severe delirium.

We determined the rate of ED transfer by year and by facility beds, with adjustment for the number of dementia beds and the availability of a 24-hour registered nurse. Differences between admission rates between the three strata were assessed using a Bayesian hierarchical model, with fixed effects for facility type (based on level of Specialist Palliative Care support), and a random cluster effect, assumed to follow a normal distribution centred on zero. Un-informative priors were used as prior distributions for all model parameters. Samples from the posterior distribution were drawn using the No U-Turn Sampler in Stan, and implemented using the brms package in R V4.0.0.

### Qualitative interviews

#### Qualitative data collection

Letters of invitation to participate in the qualitative interviews together with participant information sheets outlining the purpose of the study were provided either directly as hard copies, by post or email. Following written informed consent, perceptions of barriers and enablers to end of life care in accordance with resident wishes was sought using focus group or single interviews with aged care facility staff (assistants in nursing, registered nurses, Facility Management, nurse practitioners); general practitioners providing care for residents of participating facilities; and specialist palliative care general practitioners and nurse practitioners. Interviews were conducted late 2019 to early 2020, using an interview guide, which was adapted to each participant type (Annex A). Two female staff members conducted the focus groups and interviews over 45 to 60 min; an advanced practice nurse with an existing professional relationship with the participating residential aged care facilities and an experienced health researcher, new to the field of palliative care.

#### Qualitative data analysis

Audio files were transcribed and analysis was undertaken using NVivo. Using the qualitative descriptive approach, two members of the research team developed a code structure in advance, but adapted and coded the transcripts independently after numerous readings, using the constant comparative method [15]. Interviews ceased when no new themes emerged. The inter-rater reliability measured using Kappa statistic was 0.86 prior to thematic analysis. The final analysis and development of a theory for end of life care being delivered in line with a resident’s wish, stayed close to the data and quotes were frequently used to ensure authentic representation of participants’ words.

## Results

Of the six participating facilities, four had a registered nurse (RN) available 24 h, seven days a week. Facilities had between 19 and 29 dementia beds (accounting for 15.6–21.6% of total beds), Table 1.

**Table 1:** Facility characteristics

Facility type	Total beds	Dementia beds	24/7 RN
A. Nurse practitioner in situ	134	29 (21.6%)	1 facility
B. Outreach Specialist Palliative Care	122	19 (15.6%)	Both facilities
C. No formal Specialist Palliative Care	136	26 (19.1%)	1 facility

### Audit of medical records of deceased residents

A total of 234 medical records of deceased residents were reviewed. These deaths represented 76% of the total deaths that occurred over the study period. Due to COVID-19 visitor restrictions, 12 records were not able to be reviewed by research staff.

Of the 165 records that had a documented preferred place of care, 160 (96.7%) wished to stay in their RACF in the event of expected decline.

High coverage of advance care planning was found in facilities that did not access Specialist Palliative Care, and those utilising outreach Specialist Palliative Care (86.5% and 74.4% respectively). Facilities with NP led care appeared to have lower proportion of residents with advance care planning documented (47.8%), but this was likely due to the archived document being held off-site (missing data).

The designation of a substitute decision maker was consistently high across all facility types (range 97–100%). Documentation of a discussion with the family regarding a resident’s prognosis was highest in facilities with NP led care (91%) followed by Specialist Palliative Care outreach (88%) and no Specialist Palliative Care facilities (83%), but this was not statistically significant (p = 0.349).

Overall, 24–36% of patients experienced a delay between the identification of a symptom and symptom management in the days prior to death. The length of delay was shortest for residents in facilities with a nurse practitioner in situ compared to facilities utilising outreach Specialist Palliative Care and those not utilising Specialist Palliative Care (Table 2, p = 0.006 ). Prolonged delays beyond 24-hours ranged from 2 to > 7 days (n = 14). No prolonged delays were identified in facilities with in situ nurse practitioner.

Charts of all 25 patients who died as public hospital inpatients were reviewed. All admitted residents had a resuscitation plan and 24 (96%) had pre-emptive medications charted (the remaining resident died in the ED). There was only one documented delay in symptom management.

**Table 2:** Summary of audit of quality factors at end of life care for residents that died in the RACF

	NP insitu	Outreach Specialist Palliative Care	No formal Specialist Palliative Care	Total	p-value
Number of records reviewed	93	83	58*	234	
Average age at death	87.8	87.6	86.4	87.4	0.6
Preferred place of EOLC stay RACF	73/73 (100%)	51/51 (100%)	36/41 (87.8%)	160/165 (97%)	<0.000
Advance Care Plan (%)	43/93 (46.2%)	58 /83 (69.9%)	45/58 (77.6%)	146/234 (62.4%)	<0.000
Substitute decision maker (%)	86 (97%)	80 (98%)	58 (100%)	224 (98%)	0.133
During the last days of life:					
Documentation of a discussion with the family regarding resident’s prognosis	84 (91%)	69 (88%)	45 (83%)	198 (88%)	0.349
Of those that presented to ED in last 30 days of life, family was involved in requesting hospital transfer	7 (58.3%)	3 (16.7%)	2 (11.8%)	12 (25.5%)	0.349
Documented delay in symptom management	26/73 (36%)	21/81 (26%)	14/58 (24%)	61/212 (29%)	0.273
Length of delay < 12 h	17	3	2	22	0.006
12 to 24 h	5	5	1	11	
Days	0	8	6	14	
Delay unknown	5	5	5	15	

*12 records unable to be reviewed due to COVID-19 restrictions

### Review of ED presentations

**Fig. 1:**
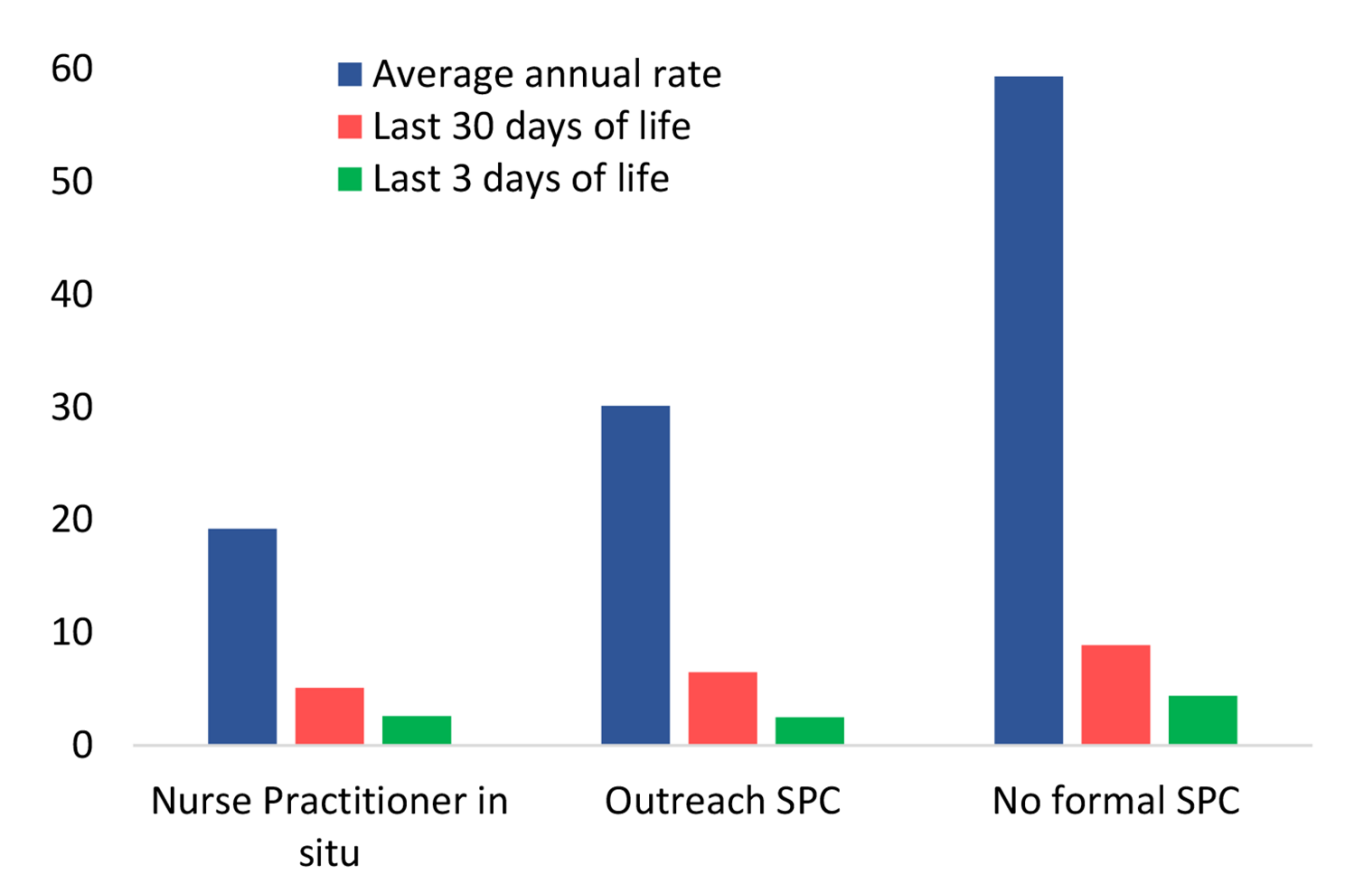
Unadjusted emergency department transfers per 100 bed-years, by year and facility type

A total of 317 presentations were reviewed. Across each time period considered, there was a consistent trend of the lowest rate of ED presentations for residents of facilities with nurse practitioners in situ, followed by facilities accessing Specialist Palliative Care outreach (Table 3; Fig. 1); the highest rates of ED presentations were in residents of facilities not accessing Specialist Palliative Care.

The difference in the annual rate of ED presentations was significantly lower for both facilities with a nurse practitioner in situ and facilities utilising outreach specialised palliative care compared to facilities not accessing Specialist Palliative Care (adjusted incident rate ratio 0.333 and 0.509 respectively, Table 3). Whilst the trend was consistent across other time points, the difference was not statistically significant. Point estimates in rates of ED transfer did not shift substantially following adjustment of key confounders, suggesting it was the level of clinical support within the facility that drove emergency department presentations rather than the presence of a registered nurse 24 h or the number of dementia beds.

**Table 3:** Unadjusted and adjusted* comparison of ED presentations by facility type

	1.Nurse practitioner in situ	2.Outreach Specialist Palliative Care	3.No formal Specialist Palliative Care	1 v 2 IRR** (95% CI)	2 v 3 IRR (95% CI)	1 v 3 IRR (95% CI)
Average annual rate (95%CI)	19.2 (2.4, 2991.5)	30.1 (2.1, 6356.5)	59.3 (7.1, 4023.6)	0.620 (0.014, 43.09)	0.526 (0.012. 17.49)	0.323 (0.017, 9.074)
Adjusted	21.5 (13.7, 33.2)	33.1 (22.6, 51.1)	65.1 (44.8, 95.4)	0.65 (0.352, 1.196)	0.509 (0.283, 0.845)	0.333 (0.174, 0.595)
Last 30 days of life	5.1 (1.7, 14.0)	6.5 (2.6, 18.2)	8.9 (3.5, 26.1)	0.770 (0.164, 3.121)	0.745 (0.171, 2.814)	0.575 (0.130, 2.01)
Adjusted	7.4 (0.02, 7277.4)	8.2 (0.01, 7277.4)	11.9 (0.69, 7124.5)	0.913 (0.006, 133.9)	0.66 (0.002, 199.9)	0.616 (0.006, 120.1)
Last 3 days of life	2.6 (0.4, 12.3)	2.5 (0.04, 14.4)	4.4 (1.4, 23.2)	1.10 (0.111, 10.367)	0.557 (0.051, 3.756)	0.596 (0.054, 3.917)
Adjusted	1.67 (0.00006, 1899.6)	1.64 (0.00008, 3397.3)	3.3 (0.00003, 4023.7)	1.05 (0.0007, 427.87)	0.53 (0.002, 238.24)	0.539 (0.001, 119.15)

*Adjusted comparison for 24 h registered nurse and number of dementia beds

** Incident rate ratio

Most ED presentations were determined to be not-avoidable (62.0%, Table 4). Facilities without a nurse practitioner had similar proportions of avoidable ED presentations (43.2% and 38.6%), and both were higher than facilities with a nurse practitioner (28.8%).

**Table 4:** Presentations categorised as avoidable or non-avoidable

	1.Nurse practitioner in situ	2.Outreach Specialist Palliative Care	3. No formal Specialist Palliative Care	Total	p-value
Avoidable	17 (28.8%)	35 (43.2%)	68 (38.6%)	120 (38.0)	0.215
Not-avoidable	42 (71.2%)	46 (56.8%)	108 (61.4%)	196 (62.0)	

*1 presentation unable to be classified & removed from this comparison

### Qualitative findings

A total of 18 focus groups or individual interviews were conducted with 54 participants (17 registered nurses, 23 assistants in nursing, 5 nurse practitioners, 9 general practitioners, of whom two were also affiliated with specialist palliative care) within a quiet space at the place of work. No participant withdrew consent.

Consistently described barriers and enablers to end of life care in aged care facilities focused on three areas: goals of care discussions and documentation; family influence; and the type of medical professional providing symptom management at end of life (general practitioner, nurse practitioner, or outreach specialist palliative care).

Theme 1. Importance of the role of family and alignment with goals of care.

Imperative to end of life care being delivered in line with a resident’s wishes was that goals of care were discussed and documented and these goals endorsed and supported by the family. Family overriding a resident’s wish to remain in their facility was the most commonly described reason for hospital transfer.

*“I see it time and time and time again, the MOLST is meaningless if the family is not aligned with the goals of care”* (Medical Order for Life Sustaining Treatment) Nurse practitioner

*“Like sometimes people ring me about a patient I don’t know and I say well what does their advanced care plan say? Because that’s what we’re meant to be following. But even then, it’s often the families that override something they’ve signed in the past.”* Visiting Medical Officer.

Underpinning this theme was the frequently reported issue of health and death literacy, which appeared to be the key reason for family overriding a resident’s end of life wishes.

“If the patient is admitted to hospital because the family requests it, it is very difficult to have a conversation about goals of care if the family are not aware that dementia is a terminal illness”, palliative care nurse practitioner.

### Theme 2. Role of palliative care provider

Participants felt where nurse practitioners were available, escalated review of deteriorating patients and symptom management was timely.

*“I find that having the nurse practitioner able to work by all of our doctors here… it happens instantly and it just flows, it’s a really good outcome for every single person*” Registered nurse

Where care was led by a General Practitioner, staff felt there were delays in the timely review of a deteriorating patient. General Practitioners reported difficulties accessing facilities after hours and having a staff member available to provide a handover and relevant information on the resident’s condition. Perceived barriers in prescribing opiates in line with Pharmaceutical Benefit Scheme indications for a patient who the General Practitioner had not seen recently (or previously at all) were also reported.

### Theme 3. Role of RACF staff

Facility staff members described a strong sense of caring for residents. Staff felt end of life care was their core business and strongly articulated their preference to provide care in place.

*“And I think trying to promote that this is our core business, and so everyone should have access to good training, good education, and good support, because, you know, everyone that’s coming into our facilities is dying… we ensure that we follow their wishes. We only get one chance to do it. We try, we do try”* Registered nurse, Aged Care Facility

Ultimately, in instances of expected deterioration, end of life care was more likely to be delivered in line with a resident’s wishes if goals of care were clearly documented and up to date and family were aligned with those goals of care. Where deterioration occurred in an unexpected manner, end of life care remained in line with resident’s wishes if goals of care were re-established in alignment with family.

## Discussion

With much criticism being targeted towards aged care facilities, it is important and timely to identify the appropriateness of care of residents and in particular the level of care provided at end of life. The findings of this mixed methods study have identified key enablers and barriers to end of life care in residents of aged care facilities.

### Preferred place of care

We demonstrate new evidence of the desire of residents of aged care facilities to largely remain in their RACF for end of life care. This finding contradicts existing evidence [1] and demonstrates the importance of context when eliciting preference of place for end of life care in the general population. When surveyed in the community and prior to life threatening illness, most people express a preference to die at home; however, once in an aged care facility and approaching end of life, the vast majority prefer to remain in situ rather than be transferred to hospital. This finding may be an important narrative in addressing public perception of experience in residential aged care. It is also important to note those residents that died during their hospital stay had quality end of life care.

### Enablers to end of life care in situ with timely symptom management

The health professional providing end of life care was a key factor in facilitating quality end of life care. We demonstrate the benefits of an integrated nurse practitioner model of care; residents of those facilities had substantially fewer ED transfers overall, and of those, a large proportion (71.2%) were unavoidable. Likewise, residents of facilities with a nurse practitioner had timelier end of life symptom management. Staff providing patient care in these facilities reported higher confidence in escalating concerns.

### Barriers to end of life care in situ with timely symptom management

Family, or substitute decision makers, overriding advance care documentation was a key barrier to end of life care being delivered in line with a resident’s wishes. This suggests the need to better orient families to advance planning concepts and optimise communication regarding irreversible deterioration. These findings were supported by the qualitative data, suggesting families were at times not understanding that death was imminent and requesting active escalated medical management of symptoms despite documented preference to stay in place. This finding was in alignment with an Australian qualitative study of perceptions of residential managers; limited health and death literacy impeded the delivery of comfort care at end of life [16].

Facilities that primarily relied on General Practitioners had more frequent ED transfers overall, and more avoidable transfers. General Practitioners described multiple influencing factors including demands of their practice, accessing facilities and quality of handover on the resident’s condition.

### Advance Care Planning/ Directive

Advance care planning is considered a key component to prepare for end of life. Whilst the presence of Advance Care Directive was the same as found in previous literature (66%),[6] in this research, the availability of such planning documentation did not appear to protect against avoidable hospital transfers or end of life care being delivered in line with wishes.

### Limitations

This study is limited by the non-random selection of participating facilities. We were also limited in our retrospective medical record review after death due to being reliant on documentation being available. These findings may have limited implications for countries with higher understanding of health and death literacy and different aged care settings.

## Conclusions

Residents overwhelmingly identify their aged care facility as their preferred place for their end of life care. This finding is critical to assessment of and resource provision to residential aged care. Integrated, responsive staff, familiar with the palliative approach are required to provide quality end of life care. Community literacy regarding residential care, advance care planning and end of life care is urgently needed. Indicators of quality of death for aged care residents are recommended to include documentation of preferred place of end of life care and the timely management of end of life symptoms.

### Electronic supplementary material

Below is the link to the electronic supplementary material.

Supplementary Material 1

## Data Availability

The datasets generated and/or analysed during the current study are not publicly available due individual privacy.

## List of abbreviations

RACF: Residential Aged Care Facility
MOLST: Medical Order for Life Sustaining Treatment
