# Supplementary material for: Preferences and end of life care for residents of aged care facilities: a mixed methods study
Source: BMC Palliat Care. 2023 Sep 1;22:124. doi: 10.1186/s12904-023-01239-9 (PMC10472708; doi:10.1186/s12904-023-01239-9)
Supplement: Supplementary file 1 — Supplementary Material 1 [file 12904_2023_1239_MOESM1_ESM.docx]

__________________________________________________________________________

| Interview guide: |
| --- |
| 1. **Welcome, introductions, reminder of taping, anonymity and expectations** |
| 1. **Experiences of end of life care within the RACF**    1. What are the usual practices when a resident is dying?    2. How are they identified?    3. Can you think back to a recent death in the facility? From your perspective, what were positive components of the death?    4. Was the death experience in line with what the resident and family wanted?   If not, what do you think hindered this from occurring?  If yes, what do you think facilitated this from occurring?   - 1. Was symptom management timely and effective?   Probe (pain addressed, no agitation, other symptoms)   - 1. Does your organisation endorse or does your practice as a clinician, routinely document the resident’s wishes for preferred place of death once they are a permanent resident in an aged care facility   2. How do you determine which family member or carer is to be contacted for a resident when they become unwell/ deteriorate?   3. How does communication and escalation occur in the facility where you work or visit if they are concerned that there is a resident deteriorating in an “expected” way and in an “unexpected” way? Who do you escalate and communicate with? |
| 1. **Palliative Care Support & framework**    1. Are you familiar with the palliative care resources or tools that are in the facility where you work or that you visit?    2. Are you aware if the facility where you work or visit regularly has a palliative care link nurse or a palliative care committee? What is your understanding of their roles?    3. Are “goals of care” and “substitute decision makers” terms commonly used in the facility where you work or visit? What other words are used? Is there a clear understanding amongst staff regarding these terms?    4. Are you aware if RACF staff have access to their own syringe drivers on site?    5. What is your understanding of the role of Specialist Palliative Care in aged care? |
| 1. **Care of residents in hospital**    1. If one of your residents is in hospital, does the hospital communicate with you about their condition? Do you have residents transferred home who are palliative? Do you receive the right support to manage this?    2. If your resident has a MOLST that says that they want palliative care during natural dying and do not want transfer to hospital, how do you decide who should be transferred to hospital? |
| 1. **Clinical Support**    1. What support do you get when a resident is dying or has died? Do you have clinical supervision, mentoring or debriefing? What support would you like? |
| 1. **General suggestions**    1. Do you have any general recommendations in mind that might improve the end of life care in a RACF?    2. Do you have any other comments? |
